# Pre-operative trichiatic eyelash pattern predicts post-operative trachomatous trichiasis

**DOI:** 10.1371/journal.pntd.0007637

**Published:** 2019-10-07

**Authors:** Emily W. Gower, Beatriz Munoz, Saul Rajak, Esmael Habtamu, Sheila K. West, Shannath L. Merbs, Jennifer C. Harding, Wondu Alemayehu, E. Kelly Callahan, Paul M. Emerson, Teshome Gebre, Matthew J. Burton

**Affiliations:** 1 Gillings School of Global Public Health, University of North Carolina at Chapel Hill, North Carolina, United States of America; 2 Wilmer Eye Institute, Johns Hopkins School of Medicine, Baltimore, Maryland, United States of America; 3 The Sussex Eye Hospital, Brighton, United Kingdom; 4 London School of Hygiene & Tropical Medicine, London, United Kingdom; 5 Helen Keller International, Dar es Salaam, Tanzania; 6 Berhan Public Health and Eye Care Consultancy, Addis Ababa, Ethiopia; 7 The Carter Center, Atlanta, Georgia, United States of America; 8 International Trachoma Initiative, Atlanta, Georgia, United States of America; 9 International Trachoma Initiative, Addis Ababa, Ethiopia; RTI International, UNITED REPUBLIC OF TANZANIA

## Abstract

**Importance:**

Trichiasis surgery programs globally have faced high rates of poor surgical outcomes. Identifying correctable risk factors for improving long-term outcomes is essential for countries targeting elimination of trachoma as a public health problem.

**Objective:**

To determine whether the location of trichiatic eyelashes prior to surgery influences development of post-operative trichiasis (PTT) within two years after surgery.

**Design:**

Secondary data analysis of four randomized clinical trials evaluating methods to improve trichiasis surgery outcomes. These include the Surgery for Trichiasis, Antibiotics for Recurrence (STAR) trial, Partnership for Rapid Elimination of Trachoma (PRET-Surgery), absorbable versus silk sutures trial, and epilation versus surgery for minor trichiasis trial.

**Setting:**

Primary trials were conducted in rural areas of Ethiopia and Tanzania

**Interventions or exposures:**

Trichiasis surgery performed with either the bilamellar tarsal rotation procedure or posterior lamellar rotation procedure

**Main outcomes:**

Prevalence of PTT within two years after surgery, location of trichiatic eyelashes pre-operatively and post-operatively

**Results:**

6,747 eyelids that underwent first-time trichiasis surgery were included. PTT rates varied by study, ranging from 10–40%. PTT was less severe (based on number of trichiatic eyelashes) than initial trichiasis for 72% of those developing PTT, and only 2% of eyelids were worse at follow up than pre-operatively. Eyelids with central only-trichiasis pre-operatively had lower rates of PTT than eyelids with peripheral only trichiasis in each of the three trials that included severe TT cases. 10% of eyelids with peripheral trichiasis pre-operatively that develop PTT have central TT post-operatively.

**Conclusions and relevance:**

Pre-operative central trichiasis is less likely than peripheral trichiasis to be associated with subsequent PTT. Regardless of type of surgery, surgeon skill levels, or pre-operative trichiasis severity, the presence of peripheral trichiasis pre-operatively is associated with higher rates of PTT. Making an incision that extends the length of the eyelid and adequately rotating the nasal and temporal aspects of the eyelid when suturing may help to minimize the chance of developing peripheral PTT.

**Trial registration:**

ClinicalTrials.gov PRET: NCT00886015; Suture: NCT005228560; Epilation: NCT00522912.

## Author summary

This manuscript reports results of an analysis of four clinical trials that were conducted to evaluate best practices for trichiasis surgery. The analysis showed that regardless of type of surgery performed or the skill level of the surgeon who conducted the surgery, similar patterns emerge. The majority of people have good surgery outcomes. Individuals who had eyelashes touching their eye only centrally before surgery were less likely to develop poor outcomes than those who had eyelashes touching at the edges of their eyelids. The findings highlight the importance of making the surgical incision the entire length of the eyelid and paying special attention to how well the edges of the eyelid are rotated at the end of surgery.

## Introduction

The global trachoma community is working to eliminate trachoma as a public health problem. Currently, 3.2 million individuals need trichiasis surgery to prevent blindness [1]. In many of the 42 trachoma-endemic countries, programs are ramping up to meet this surgical demand. Post-operative trichiasis (PTT) is defined as one or more eyelashes touching the globe or evidence of epilation following surgery. It incorporates both surgical failure, in which the eyelid was never adequately corrected, and recurrent trichiasis, which manifests from ongoing disease progression. This term was defined during discussions at the Second Global Scientific Meeting on Trachomatous Trichiasis [2]. PTT undermines efforts to eliminate trachoma and historically has been problematic in many programmatic settings; surgical quality needs to be improved in order for programs to meet their surgical targets in an effective way. Experts recognize that trichiasis surgery will be needed for many years after the prevalence of active trachoma, characterized by trachomatous inflammation follicular (TF), declines below the elimination threshold of 5% among children aged <10 year, and it is critical to identify best surgical practices right away.

Previous studies have demonstrated an association between surgical skill and PTT rates. Within a given program, PTT rates often vary by surgeon [3–7]. Additionally, some evidence shows that shorter incisions lead to higher PTT rates [8,9]. This study aimed to determine what additional factors predict PTT, and whether these factors are consistent across surgical procedures. Specifically, we aimed to determine whether PTT occurs in the same segment of the eyelid as pre-operative trichiasis and whether the same patterns are observed across surgical procedures and surgeon skill levels. These data may help trichiasis surgery experts to evaluate current surgical techniques and identify ways to improve surgical outcomes.

## Methods

### Data sources

We utilized data from four clinical trials aimed at improving trichiasis surgery outcomes that gathered data on pre- and post-operative trichiatic eyelash locations and trichiasis severity. The Surgery for Trichiasis, Antibiotics for Recurrence (STAR) trial was conducted in Ethiopia between 2002–2007 to evaluate whether single-dose oral azithromycin at the time of trichiasis surgery reduces PTT risk as compared to six weeks of topical tetracycline use [6,10]. The best three surgeons in the region were selected to serve as the trial surgeons. These surgeons were selected based on assessment of all surgeons in the region by an external expert, utilizing the World Health Organization’s Final Assessment of Trichiasis Surgeons guidelines [11]. The Partnership for Rapid Elimination of Trachoma (PRET) Surgery trial was conducted in Tanzania between 2009–2012. The trial compared surgical outcomes for eyelids operated with standard BLTR instrumentation versus surgery performed with the TT clamp [5,12]. All surgeons working in the Mtwara or Lindi regions were invited to participate, and 18 surgeons conducted trial-related surgeries. Each surgeon was assigned to utilize only one type of instrumentation throughout the trial.

These trials utilized similar data collection methods and definitions. Detailed methods have been reported previously [5,10,13]. Briefly, all participants received trichiasis surgery using the bilamellar tarsal rotation procedure (BLTR) [14]. In the STAR trial, all surgeries were performed using artery forceps and a lid plate. In PRET, half of the surgeries were performed with artery forceps and a lid plate, and the other half were performed with the TT clamp [12]. Participants were followed at 2 weeks, 6 weeks, 1 year and 2 years. At baseline and each follow-up visit, a certified examiner masked to the treatment assignment evaluated the presence of trichiatic eyelashes and epilation and recorded the number and location of trichiatic eyelashes. We divided the eyelid into equal thirds and recorded the location(s) as nasal, central, and/or temporal. We defined the location of trichiatic eyelashes as the location of the follicle from which these eyelashes were emanating. In the STAR trial, epilation was recorded as present or absent, while in the PRET Surgery trial extent of epilation was categorized into <10 eyelashes epilated, 10–20 eyelashes epilated or >20 eyelashes epilated based on eyelash follicles or visible eyelash stubs, [5] which translates to <1/3, 1/3-2/3, and >2/3 of the eyelid epilated. In STAR, only one eye per participant was enrolled, while in PRET if both eyes had TT and were previously unoperated, both eyes were enrolled.

Two concurrent randomized trials were conducted using the posterior lamellar tarsal rotation procedure (PLTR), a modification of the Trabut procedure [14]. The first PLTR trial, henceforward called the “Suture trial”, evaluated whether outcomes differed for eyelids operated with absorbable Vicryl suture compared with eyelids operated with 4–0 silk suture [15]. The trial enrolled patients who had major trichiasis, defined as 6+ trichiatic eyelashes. Concurrently, patients with minor trichiasis, defined as 5 or fewer trichiatic eyelashes, were recruited for a trial, henceforth, referred to as the “Epilation trial,” [16] to compare two-year visual outcomes in eyes operated with PLTR compared with eyes where epilation was recommended and high-quality epilation forceps and epilation training were provided. These two trials occurred simultaneously, utilized the same surgeons, and were conducted in the same villages in Ethiopia’s Amhara region. All surgeries were performed with a Trabut plate. In each trial, only one eyelid was enrolled for the purposes of analysis; the second eyelid was treated in the same way, unless the participant requested different treatment for the non-study eyelid. Participants were followed-up every six months for two years. For the current analyses, only those eyes that received surgery were included; all eyelids in the epilation arm were excluded.

As in the BLTR trials, in the Suture and Epilation trials we collected data on the presence of trichiatic eyelashes and epilation and recorded the number and location of trichiatic eyelashes at each study visit. However, the definition of central trichiatic eyelashes differed slightly from that of the BLTR trials. Central/corneal eyelashes were those that were seen to touch the cornea in the primary position of gaze, irrespective of the location of the eyelash follicle. As the average corneal width is slightly more than one-third of the width of the upper eyelid, central eyelashes made up a slightly greater proportion of the eyelid in the PLTR trials as compared to the BLTR trials. For all four trials, epilation was defined as the presence of broken or newly growing eyelashes, or areas of absent eyelashes.

Trichiasis severity was defined based on number of trichiatic eyelashes and presence of epilation. For the PRET, Suture and Epilation trials, we used the definition previously reported in the PRET trial, [5] which takes into account the extent of epilation. In the STAR trial, epilation was recorded simply as present or absent, so a slightly modified definition was used (Table 1), recognizing that this definition may understate trichiasis severity. Although the precise definition varied by trial, the intent was to categorize those with <5 trichiatic eyelashes (present or epilated) as mild, 5–9 as moderate and 10+ as severe.

**Table 1:** Trichiasis severity classification by trial.

Category	STAR	PRET, Suture, Epilation
**Mild**	1–4 trichiatic eyelashes and no epilation OR Epilation and no trichiatic eyelashes	1–4 trichiatic eyelashes and no epilation OR <1/3 of eyelid epilated and no trichiatic eyelashes
**Moderate**	5–9 trichiatic eyelashes and no epilation OR 1–4 trichiatic eyelashes and epilation	5–9 trichiatic eyelashes and no epilation OR 1–4 trichiatic eyelashes and <1/3 epilated
**Severe**	5–9 trichiatic eyelashes and epilation OR 10+ trichiatic eyelashes	5–9 trichiatic eyelashes and any epilation OR 10+ trichiatic eyelashes OR >1/3 eyelid epilated

### Ethics approval

Each trial followed the tenets of the Declaration of Helsinki, and each received institutional and in-country ethics approval prior to recruiting participants. Specifically, the Johns Hopkins Medical Institutions Review board approved the STAR and PRET trials. The London School of Hygiene and Tropical Medicine Ethics Committee and the Emory University Institutional Review Board approved the Suture and Epilation Trials. PRET-Surgery received in-country approval from the National Institute for Medical Research (NIMR) in Tanzania. The STAR, Suture, and Epilation Trials received in-country approval from the National Health Research Ethics Review Committee of the Ethiopian Ministry of Science and Technology. The three trials conducted since the creation of clinicaltrials.gov were registered on that site (PRET: NCT00886015; Suture: NCT005228560; Epilation: NCT00522912). Additionally, the Johns Hopkins Medical Institutions and the Wake Forest School of Medicine institutional review boards approved the current analyses. All participants provided written informed consent prior to participation.

### Analyses

We included all study eyes that received surgery. All analyses were conducted at the trial level; we did not combine datasets given that we wanted to compare outcomes across procedures and trichiasis severity levels. We used frequencies to determine PTT presence at each follow-up interval. While we present all possible eyelash patterns at baseline, we combined nasal and temporal eyelashes into “peripheral” for follow-up analyses, because of the strong similarity of findings between these groups. For analyses utilizing follow-up data, we characterized eyelids based on the first follow-up visit at which PTT was present. If an individual had no trichiasis at one visit, followed by a report of repeat surgery at the next visit, the eyelid was assumed to have PTT that developed during the intervening timeframe. Cumulative two-year PTT rates are based on PTT presence at one or more follow up visits. We used Kaplan-Meier product limit estimates to estimate the cumulative PTT rate, accounting for censoring of observations when appropriate.

We used logistic regression to evaluate the association between baseline eyelash location and odds of developing PTT. We used generalized estimating equations to adjust for the correlation between two eyes of an individual and adjusted these analyses for baseline number of trichiatic eyelashes and treatment assignment. In the PRET trial, since either the TT clamp or artery forceps and a lid plate were used, we also adjusted for the type of instrumentation in the multivariate analysis. To isolate the effects of central versus peripheral eyelashes, we limited this regression analysis to individuals with only eyelashes touching centrally or peripherally, with no evidence of epilation.

## Results

### Baseline characteristics

**Fig 1:**
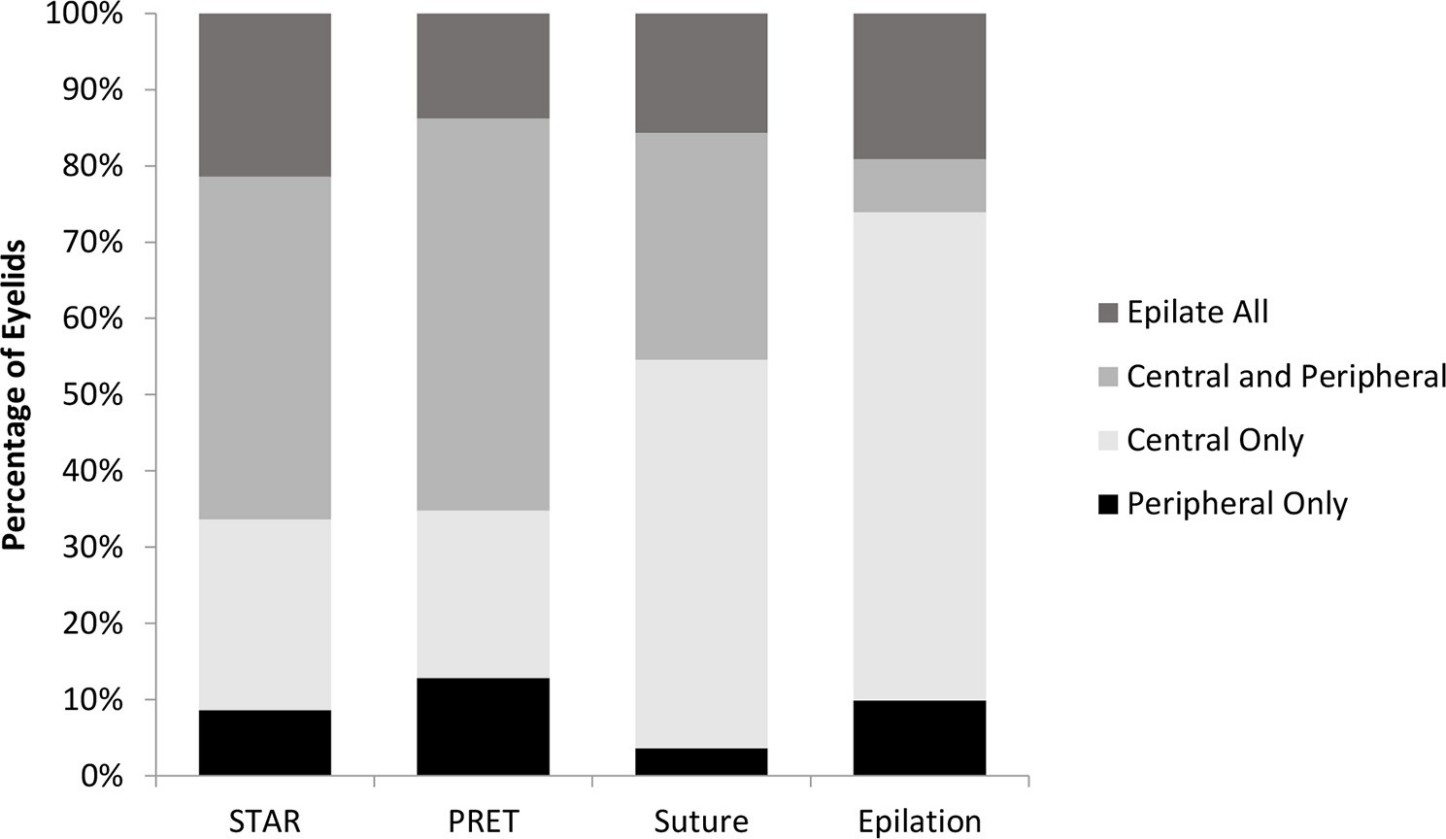
Location of trichiatic eyelashes pre-operatively by trial (see attached).

A total of 6,747 eyelids were included. The pre-operative trichiasis severity differed across trials, as per the eligibility criteria, with the epilation trial having mostly mild and moderate trichiasis cases, while the remaining trials had 40–57% with severe trichiasis pre-operatively. Nearly 60% of eyelids had evidence of epilation, and 15–20% of eyelids in each trial had all trichiatic eyelashes epilated. Eyelids had similar pre-operative trichiatic eyelash patterns in PRET and STAR, with 22–25% having central-only trichiasis. In the Suture and Epilation trials, a larger percentage of eyelids had central/cornea touching involvement, reflecting the different definitions used (Table 2; Fig 1). In each study, among eyelids with only one location involved, the most common location was central, followed by temporal, and then nasal.

**Table 2:** Baseline characteristics of eyelids enrolled in each trial, n(column %).

Characteristic	STAR	PRET	Suture	Epilation
**Participant-level Characteristics**				
**Number of Participants**	1452	1917	1300	650
Female	1121 (77.2)	1427 (74.4)	1015 (78.1)	448 (68.9)
Age group				
<50 years	694 (47.8)	634 (33.1)	614 (47.2)	296 (45.5)
50–59 years	384 (26.5)	494 (25.8)	346 (26.6)	173 (26.6)
60–69 years	321 (22.1)	501 (26.1)	245 (18.9)	116 (17.9)
≥70 years	53 (3.6)	288 (15.0)	95 (7.3)	65 (10.0)
Age (mean(sd))	48.9 (13.0)	55.2 (13.3)	49.8 (13.2)	49.9 (14.4)
**Eye-level Characteristics**				
Number of eyelids	1452	3345	1300	650
Left eyelids	735 (50.6)	1697 (50.7)	627 (48.2)	331 (50.9)
Trichiatic Eyelashes:				
None (epilate all)	309 (21.3)	460 (13.8)	204 (15.7)	124 (19.1)
1–4	522 (36.0)	1177 (35.2)	476 (36.6)	504 (77.5)
5–9	239 (16.5)	703 (21.0)	355 (27.3)	22 (3.4)
10+	380 (26.2)	1000 (29.9)	265 (20.4)	0 (0.0)
Epilation:				
None	467 (32.2)	1643 (49.1)	248 (19.1)	303 (46.6)
Some epilation, trichiatic eyelashes present also	674 (46.4)	1242 (37.1)	848 (65.2)	223 (34.3)
All trichiatic eyelashes epilated	309 (21.3)	460 (13.8)	204 (15.7)	124 (19.1)
Trichiasis Severity:				
Mild	503 (34.7)	650 (19.4)	123 (9.5)	416 (64.0)
Moderate	365 (25.2)	876 (26.2)	438 (33.7)	220 (33.9)
Severe	582 (40.1)	1819 (54.4)	739 (56.8)	14 (2.1)
Location of Trichiatic Eyelashes*†:				
Epilate all	309 (21.3)	460 (13.8)	204 (15.7)	124 (19.1)
Nasal only	21 (1.5)	163 (4.9)	9 (0.7)	22 (3.4)
Central only	361 (24.9)	742 (22.2)	658 (50.6)	412 (63.4)
Temporal only	96 (6.6)	189 (5.6)	38 (2.9)	38 (5.9)
Nasal & central	68 (4.7)	537 (16.1)	103 (7.9)	28 (4.3)
Nasal & temporal	8 (0.5)	76 (2.3)	0 (0.0)	4 (0.6)
Central & temporal	202 (13.9)	572 (17.1)	253 (19.5)	21 (3.2)
Nasal, central & temporal	384 (26.5)	604 (18.1)	35 (2.7)	1 (0.1)

Notes: PRET—Trichiatic eyelashes present but number missing for 3 eyes; 2 eyes missing epilation status.

STAR—2 eyelids missing number and location of trichiatic eyelashes; 1 additional eyelid missing location only.

* Epilation may also be evident for any of the eyelash locations

†Remaining analyses combine nasal and temporal into “peripheral”

### Follow-up characteristics

Follow up was excellent, with each study reporting >90% follow up (STAR, PRET and Epilation each 98%, Suture 94%). At follow up, the majority of eyelids had no trichiasis, with two-year PTT rates ranging from 10–40% across studies (Tables 3 and 4). The STAR trial, which used the most qualified surgeons available, had the lowest two-year PTT rate, and the PRET trial, which utilized a broad range of surgeons with varying skill levels, had the highest rate. For all studies, the majority of PTT cases developed between the three-month visit and one year (Table 3).

**Table 3:** Frequency and characteristics of post-operative trichiasis overall and by visit.

	STAR	PRET	Suture	Epilation
Number of eyes	1452	3345	1285	641
Total number of PTT	136	1333	441	114
% Post-operative Trichiasis (PTT) Cumulative to Two Years*	9.5	40.3	34.7	18.0
**New PTT among the eyes at risk (%)**†				
≤3 months	32/1452(2.0)	196/3343 (5.9)	138/1285 (10.7)	NA (no visit)
>3months—1 year	75/1401 (5.4)	894/3121 (28.6)	214/1131 (18.9)	75/635 (11.8)
>1 year—2 years	29/1289 (2.3)	243/2193 (11.1)	89/889 (10.0)	39/539 (7.2)
**Number of Trichiatic Eyelashes at First Visit with PTT (%)**^**§**^				
0 (epilate all)	3 (2.3)	56 (4.2)	95 (22.4)	17 (15.7)
1–2	80 (60.6)	732 (55.4)	239 (56.4)	83 (76.9)
3–5	32 (24.2)	245 (18.6)	43 (10.1)	5 (4.6)
6–9	16 (12.1)	182 (13.8)	28 (6.6)	1 (0.9)
10+	1 (0.8)	105 (8.0)	19 (4.5)	2 (1.9)
Epilation at first visit with PTT	20 (14.7)	94 (7.1)	136 (32.1)	218 (19.4)
**Trichiasis Severity at First Visit with PTT (%)**^**§**^				
Mild	117 (88.0)	893 (67.7)	337 (79.5)	100 (92.6)
Moderate	10 (7.5)	207 (15.7)	58 (13.7)	6 (5.6)
Severe	6 (4.5)	220 (16.7)	29 (6.8)	2 (1.9)
**Location of Trichiatic Eyelashes at First Visit with PTT (%)**				
Epilate all	3 (2.2)	56 (4.2)	95 (22.4)	17 (15.7)
Peripheral^‡^ only (+/- epilation)	68 (50.4)	511 (38.7)	80 (18.9)	25 (23.2)
Central only (+/- epilation)	43 (31.9)	487 (36.9)	193 (45.5)	61 (56.5)
Central and peripheral	21 (15.6)	266 (20.2)	56 (13.2)	5 (4.6)

*Post-operative trichiasis (PTT) at 1+ visit, regardless of whether it was present at subsequent visits. Kaplan-Meier product limit estimates used to estimate the cumulative rate to two years, censoring observations when appropriate.

†Participants permanently lost to follow-up excluded from the denominator for the relevant periods. If they were temporarily lost to follow up but seen at a future visit, they are included in the denominator.

‡ Nasal and/or temporal location(s)

§Missing data: 13 PRET, 17 Suture and 6 epilation study eyelids had surgery between visits; hence, eyelash-level information at time of post-operative trichiasis is not available. PRET: 1 additional eyelid missing epilation status. STAR: 3 eyelids missing number of eyelashes, 2 of which have location indicated. Denominators adjusted to reflect missing data.

**Table 4:** Baseline eyelash location(s) and post-operative trichiasis (PTT) to cumulative to two years*.

	Two-year Post-operative Trichiasis n, (%)											
Baseline Location of Trichiatic Eyelashes	STAR			PRET			Suture			Epilation		
	N eyes	PTT	p-value	N eyes	PTT	p-value	N eyes	PTT	p-value	N eyes	PTT	p-value
Epilate all	311	34 (7.9)	0.02	460	149 (32.4)	<0.001	199	72 (36.2)	<0.001	123	11 (8.9)	<0.001
Peripheral only, no epilation	63	5 (9.6)		199	69 (34.7)		8	3 (37.5)		32	4 (12.5)	
Peripheral only and evidence of epilation	62	7 (11.3)		229	106 (46.3)		39	13 (33.3)		29	11 (37.9)	
Central only, no epilation	181	3 (1.7)		331	75 (22.7)		124	25 (20.2)		243	34 (14.0)	
Central only and evidence of epilation	179	16 (8.9)		411	96 (23.4)		528	151 (28.6)		166	30 (18.7)	
Central and peripheral, no epilation	223	27 (12.1)		1111	555 (50.0)		112	51 (45.5)		23	8 (34.8)	
Central and peripheral and evidence of epilation	431	44 (10.2)		600	282 (47.0)		275	126 (45.8)		25	16 (64.0)	

Notes: STAR trial based on 1450 eyelids because 2 are missing lashes location at baseline exam. PRET trial based on 3341 because 2 are missing eyelash location at baseline exam and 2 lost to follow-up after surgery. Suture trial based on 1285 eyelids, because 15 eyelids were lost to follow-up after surgery. Epilation trial based on 641 eyelids because 9 eyelids were lost to follow-up after surgery. P<0.05 indicates that a significant difference in rates of PTT across locations within a trial.

*Based on the first visit with PTT.

Among 1,985 eyelids with PTT with complete information, disease was typically less severe than it had been pre-operatively, with 72% of PTT cases having less severe disease post-operatively (Table 5). A minority of eyelids (n = 40; 2%) had more severe PTT at the first follow up where trichiasis was present than just prior to surgery. Among eyelids with PTT, epilation was much less common at follow up than at baseline, with only 14% of eyelids with PTT showing evidence of epilation compared with 60% at baseline.

**Table 5:** Change in trichiasis severity before and after surgery among eyelids that developed post-operative trichiasis.

		Trichiasis Severity Change		
Study	N	Better	Same	Worse
PRET	1320	71%	27%	2%
STAR	133	68%	26%	7%
Suture	424	81%	17%	1%
Epilation	108	48%	49%	3%
Total	1985	72%	26%	2%

### Association between pre-operative trichiasis and PTT

**Fig 2:**
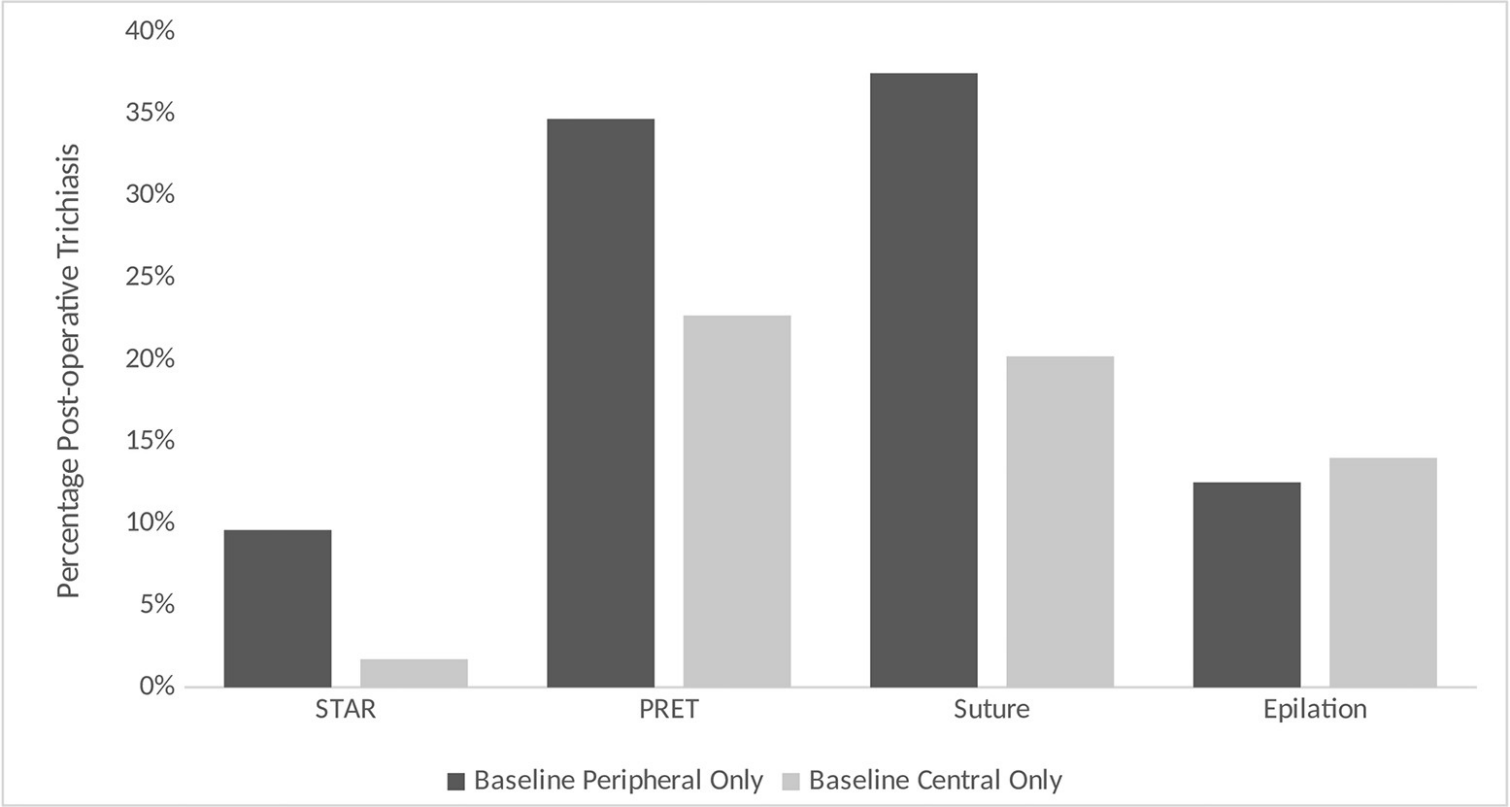
Percent post-operative trichiasis by baseline location among eyes with peripheral only or central only trichiasis and no epilation, by study (see attached).

Eyelids with trichiatic eyelashes in >1 location pre-operatively were the most likely to develop PTT (Table 4). Among the eyelids with either peripheral-only or central-only trichiatic eyelashes pre-operatively and no epilation, PTT was more common in eyelids with peripheral trichiasis pre-operatively, and this association was seen across all three studies that included severe TT patients (Fig 2). After adjusting for baseline number of trichiatic eyelashes and age, a statistically significant association was seen between pre-operative peripheral eyelashes and increased PTT risk for the PRET and STAR trials (Table 6). For simplicity of interpretation, we excluded eyelids with epilation from the modeling analysis; however, including those eyelids in the analysis resulted in similar findings (S1 Table).

**Table 6:** Association between baseline trichiatic eyelash location and post-operative trichiasis among eyelids with no evidence of epilation at baseline.

Study	N of eyes with central only or peripheral lashes only & no epilation at baseline	Post-operative Trichiasis N(%)	Factor	Odds Ratio* (95% CI)	p-value
STAR	244	8 (3.3)	Central	1.00	0.04
			Peripheral	4.81 (1.09–21.27)	
PRET	530	144 (27.1)	Central	1.00	0.006
			Peripheral	1.81 (1.18–2.78)	
Suture	132	28 (21.2)	Central	1.00	0.14
			Peripheral	3.21 (0.68–15.15)	
Epilation	275	38 (13.8)	Central	1.00	0.71
			Peripheral	0.81 (0.26–2.49)	

*Adjusted for number of trichiatic eyelashes at baseline and age, PRET is also adjusted to account for treatment assignment (surgical instrument).

### Location of pre-operative trichiasis and PTT

**Fig 3:**
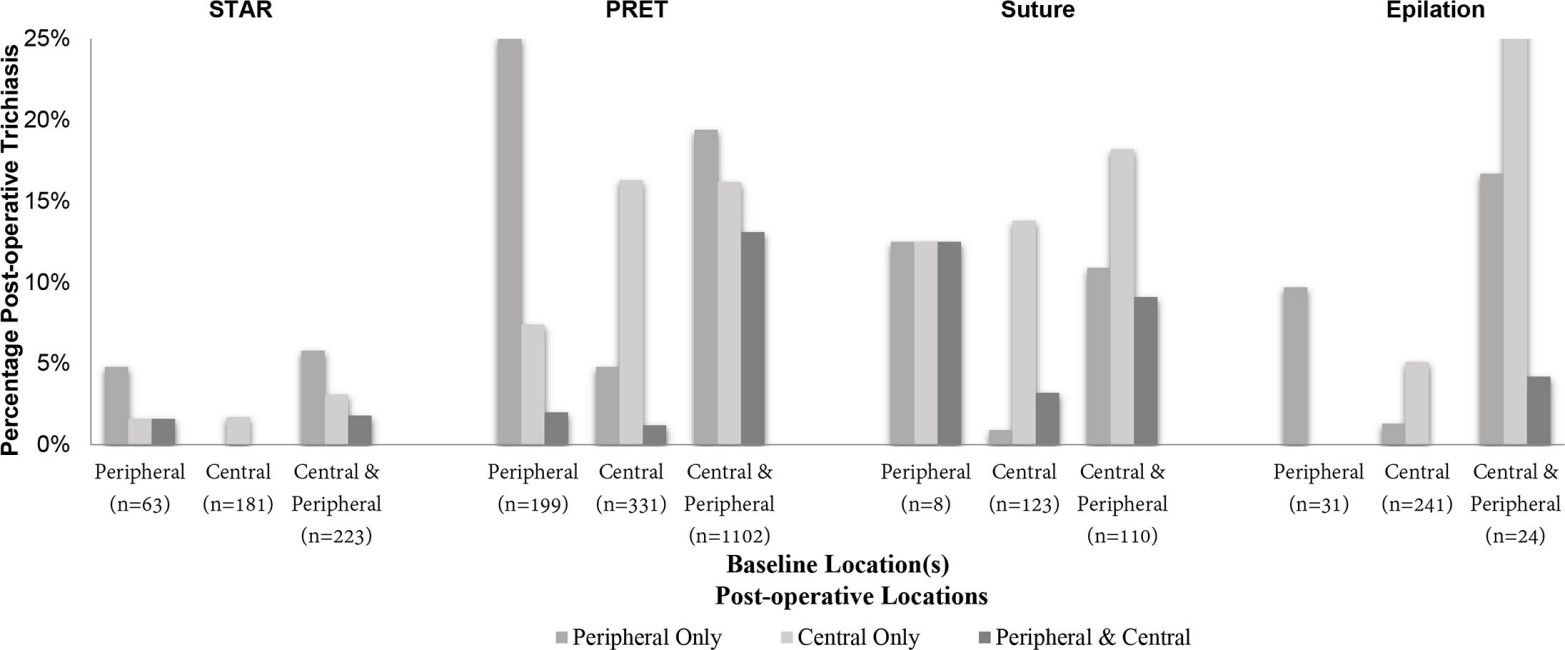
Prevalence of post-operative trichiasis (PTT) and post-operative trichiatic eyelash location, according to pre-operative eyelash location among eyelids with no epilation, by study (see attached).

For each trial and each baseline eyelash-location pattern in eyelids with no epilation, Fig 3 shows the percent of eyelids with PTT, by PTT location. Most commonly, eyelashes occurred in the same location as before. For example, in PRET 199 eyelids had peripheral-only TT with no epilation at baseline, and 25% of these eyelids had peripheral TT at follow up. However, sometimes the location changed. For example, among the same 199 eyelids from PRET, 19 (10%) had trichiatic eyelashes centrally at the first visit where PTT was reported. A similar proportion of eyelids with central only TT at baseline had peripheral-only TT at follow up. Among 1,102 PRET eyelids with central and peripheral trichiasis at baseline, the most common location for PTT was peripheral-only, showing a decrease in the TT severity.

## Discussion

This study analyzed data from four large, randomized clinical trials to provide a robust analysis that demonstrated an association between pre-operative trichiatic eyelash location(s) and PTT risk. From a programmatic viewpoint, the study findings are optimistic. Central trichiasis is most likely to cause corneal opacity and blindness, and eyelids with central-only trichiasis pre-operatively were less likely to develop PTT. Conversely, we found that eyelids with peripheral-only trichiasis pre-operatively were significantly more likely to have PTT than eyelids with central-only trichiasis. This finding was consistent across all three studies of patients with severe trichiasis, in different settings that utilized surgeons with a variety of skill levels and two different procedures. The findings highlight the need for programs to pay particular attention to patients with peripheral trichiasis to ensure that the surgeon properly corrects the eyelid in order to minimize the risk of peripheral PTT.

### Peripheral eyelashes are more likely to result in PTT

The most likely explanation for the finding that peripheral baseline trichiasis is an important risk factor for PTT is that peripheral eyelashes are at more risk of insufficient rotation during surgery, which may occur for several reasons. First, the incision may not extend all the way through the nasal and temporal aspects of the eyelid. This may happen because the surgeon is concerned about increasing bleeding by making a wider incision and/or cutting through the eyelid margin. Further, if the hemostats are positioned too close together or an eyelid clamp that is too small is used, the incision will be shorter than the eyelid width. Shorter incisions have been associated with a significant increase in PTT [8,9]. When surgery is performed with a short incision, the peripheral aspects of the eyelid will not be fully incised. As no rotation sutures are placed in the un-incised parts of the eyelid, under-correction of the eyelid peripherally is likely to result.

Another explanation is that the nasal aspect of the eyelid is inherently more difficult to rotate because of the shorter height of the tarsus nasally. Even if the incision length is adequate, there is less proximal tarsus to which the distal fragment can be stabilized. Thus, under-correction is more likely to happen nasally, regardless of whether or not the incision length is adequate. Two studies that have analyzed immediate post-operative photographs to evaluate the eyelid correction have shown that eyelids with under-correction are more likely to develop PTT [9,17]. One of these studies also investigated the association between baseline eyelash locations and PTT, [9] and it reports findings consistent with the current study.

The challenges of surgically addressing peripheral eyelashes should be taught at the programmatic level both during surgeon training and supportive supervision visits. Country programs should inform their surgeons that even in situations with low PTT rates, peripheral TT is more likely to result in PTT; therefore, the importance of checking the incision length carefully before placing sutures should be emphasized. Shorter incisions can be extended with scissors after the hemostats or clamp is removed. Similarly, once the surgeon places sutures, the surgeon should double check the peripheral eyelid aspects to ensure that they are adequately rotated, similar to the central portion. Additionally, training programs may want to increase emphasis on what defines adequate rotation of the eyelid nasally and how to achieve that rotation. Since limited images of adequately rotated eyelids are provided as part of training packages, we developed a card specifically addressing peripheral rotation and its effects (appendix).

### Correlation between pre-operative and post-operative trichiasis

Given that in most instances, PTT returns to the same location as incident trichiasis, surgeons must be aware of the trichiasis location pre-operatively so that they can be particularly attentive to this area and confirm that the region with trichiasis is slightly over-rotated at the completion of surgery. This study also showed that the location of post-operative trichiasis may differ from presenting trichiasis. As central eyelashes are a greater risk factor for corneal blindness [18], it is fortunate that only about 10% of eyelids with peripheral-only eyelashes pre-operatively that develop PTT have central trichiasis post-operatively. The development of new central PTT may result from poor incision placement, incision length or suture tension causing variable rotation in different areas of the eyelid.

The majority of eyelids with PTT had less severe disease at follow up than they did pre-operatively, suggesting that while the surgery was not completely successful in all cases, it generally improved the eyelid status and reduced the trichiatic eyelash burden. This was consistent across procedures and surgeon skill levels. This finding is important, as programs look to evaluate the benefits of conducting surgery and face questions regarding the cost-benefit of surgery. A small number of eyelids (n = 40) in this study had more severe trichiasis at follow up than they did pre-operatively. In most instances, these eyelids were categorized as having mild TT pre-operatively and moderate PTT at follow up, although seven changed from mild disease pre-operatively to severe disease post-operatively.

When considering the findings from this study, it is important to note that we analyzed follow up data strictly from the first visit where PTT was present. In some cases, PTT could progress over time. However, we wanted to look at the impact of surgery on surgical outcomes, and the first visit with PTT provides the best opportunity to do so.

### Limitations

This secondary data analysis has limitations. The PLTR trials utilized a different method for categorizing trichiatic eyelash locations than the BLTR trials. This difference likely results in an increase in the proportion of eyelids with central-only trichiatic eyelashes in the PLTR studies, as seen both at baseline (Table 2) and follow up (Table 3). The data suggest a stronger association of PTT with peripheral TT in the BLTR trials than in the PLTR trials. With the available data, it is difficult to determine whether the reduced association between peripheral TT eyelashes and PTT in the PLTR trials results from the smaller segments classified as peripheral, or whether the difference is due to the different surgical techniques used between these trials. Despite this difference, we found that peripheral-only trichiatic eyelashes resulted in higher PTT rates in all three studies that included patients with severe TT, which suggests that peripheral eyelashes likely have the biggest impact on PTT.

Additionally, the STAR trial did not collect extent of epilation, which likely results in the severity categorization being slightly more mild than the other trials. However, findings involving severity were consistent across trials, which suggests that this difference had a limited impact. Finally, we restricted our follow up location analyses to eyelids that did not have evidence of epilation at baseline or follow up in order to get the most accurate picture of eyelash patterns by minimizing misclassification. Sensitivity analyses including eyelids with epilation did not change the clinically-significant interpretations. Thus, the primary impact of excluding these eyelids is a reduction in sample size.

### Summary

This analysis demonstrated that across trichiasis surgery procedures, surgeon skill levels, and trichiasis severity levels, peripheral trichiatic eyelashes are the most likely to result in PTT. Utilizing data from four trials with varying surgeon skill levels and surgical procedures allows for a robust analysis of these factors. This finding emphasizes the importance of trichiasis-surgery training programs focusing on ensuring adequate incision length and appropriate correction of peripheral eyelashes.

## Supporting information

S1 Table Association between baseline trichiatic eyelash location and post-operative trichiasis among eyelids with central only or lateral only eyelashes, irrespective of epilation at baseline. (DOCX)

S1 Fig Good and inadequate rotation of peripheral lashes. (TIF)

S2 Fig Components of achieving good rotation of peripheral lashes. (TIF)
