# Supplementary material for: Pre-operative trichiatic eyelash pattern predicts post-operative trachomatous trichiasis
Source: PLoS Negl Trop Dis. 2019 Oct 7;13(10):e0007637. doi: 10.1371/journal.pntd.0007637 (PMC6797216; doi:10.1371/journal.pntd.0007637)
Supplement: S1 Table — (DOCX) [file pntd.0007637.s001.docx]

**S1. Table: Association between Baseline Trichiatic Eyelash Location and Post-operative Trichiasis among Eyelids with Central Only or Lateral Only Eyelashes, Irrespective of Epilation at Baseline**

| **Study** | **N of eyes with central only or peripheral lashes only** | **Post-operative Trichiasis**  **N(%)** | **Factor** | **Odds Ratio***  **(95% CI)** | **p-value** |
| --- | --- | --- | --- | --- | --- |
| STAR | 485 | 31 (6.4) | Central | 1.00 | 0.12 |
|  |  |  | Peripheral | 1.81 (0.85 – 3.89) |  |
| PRET | 1170 | 346 (29.6) | Central | 1.00 | 0.002 |
|  |  |  | Peripheral | 1.60 (1.25 – 2.05) |  |
| Suture | 699 | 192 (27.5) | Central | 1.00 | 0.76 |
|  |  |  | Peripheral | 1.12 (0.55 – 2.26) |  |
| Epilation | 470 | 79 (16.8) | Central | 1.00 | 0.09 |
|  |  |  | Peripheral | 1.75 (0.91 – 3.36) |  |

*Adjusted for number of trichiatic eyelashes at baseline and age, PRET is also adjusted to account for treatment assignment (surgical instrument).
