# Supplementary figures and images for: Pre-operative trichiatic eyelash pattern predicts post-operative trachomatous trichiasis
Source: PLoS Negl Trop Dis. 2019 Oct 7;13(10):e0007637. doi: 10.1371/journal.pntd.0007637 (PMC6797216; doi:10.1371/journal.pntd.0007637)

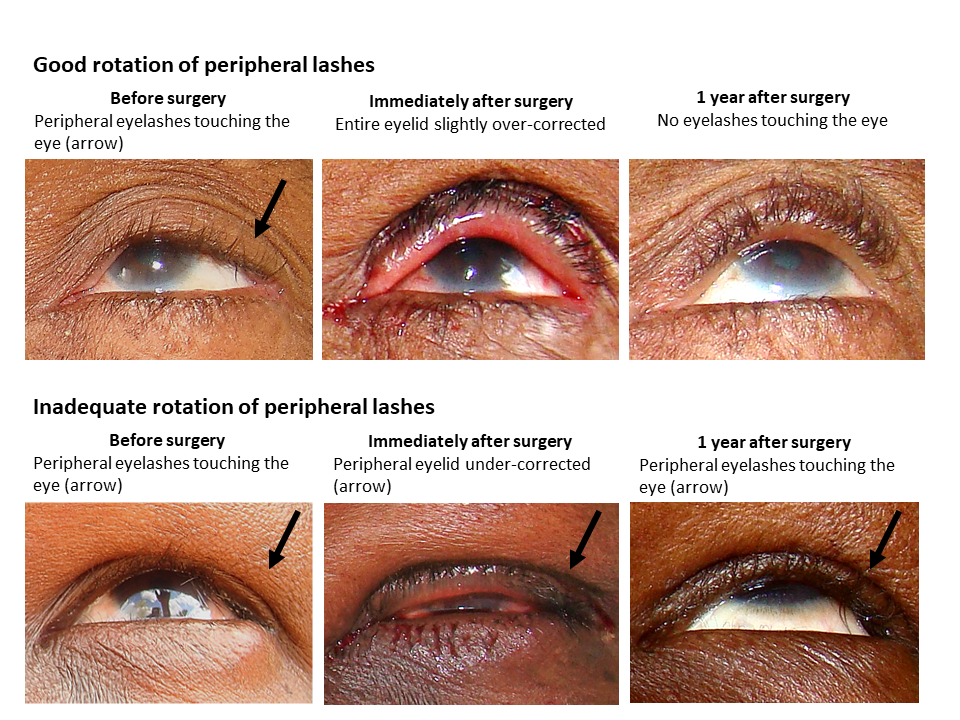

Supplement: S1 Fig — (TIF) [file pntd.0007637.s002.TIF]

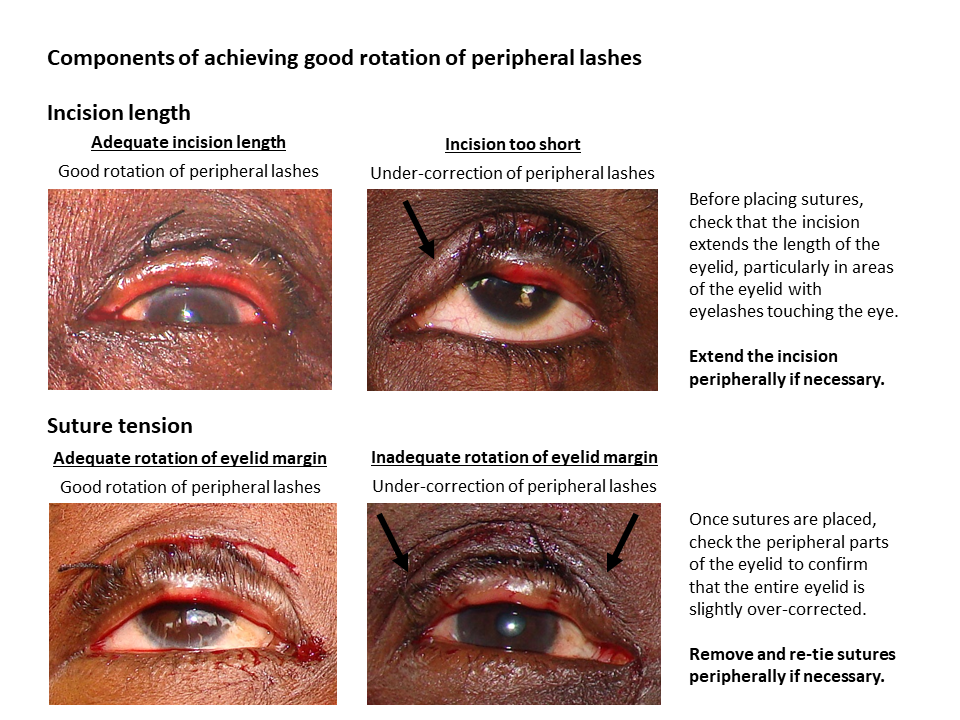

Supplement: S2 Fig — (TIF) [file pntd.0007637.s003.TIF]
